# Effects of an App-Based Physical Exercise Program on Selected Parameters of Physical Fitness of Females in Retirement: A Randomized Controlled Trial

**DOI:** 10.3389/fphys.2022.821773

**Published:** 2022-03-04

**Authors:** Sonja Jungreitmayr, Christina Kranzinger, Verena Venek, Susanne Ring-Dimitriou

**Affiliations:** ^1^Department of Sport and Exercise Science, Paris Lodron University of Salzburg, Salzburg, Austria; ^2^Salzburg Research Forschungsgesellschaft mbH, Salzburg, Austria

**Keywords:** active aging, digital, strength, AAL, healthy aging, flexibility

## Abstract

Modern technologies enable new options in the delivery of physical exercise programs. Specially designed app-based programs can be used to help older people in particular to integrate physical exercise into their daily lives. This study examines the influence of an app-based physical exercise program on selected parameters of physical fitness, such as muscular strength, balance, and flexibility. The women (*n* = 110) were on average 65.3 (± 1.5) years old and, compared to age-specific norm values, healthy. The 14-week intervention consisted of an app-based, unsupervised physical exercise program, in which the exercise frequency and duration of sessions were self-selected. The physical exercise program consisted of simple, functional exercises such as arm circles, squats, lateral raises. The participants were provided with an elastic resistance band and an exercise ball allowing them to increase exercise intensity if needed. Participants were randomly assigned to intervention group (IG) and control group (CG). 71% of the IG used the physical exercise program at least 1.2 times per week, whereas 25% of the IG showed usage rates above four times per week. Significant effects were found in the domains of muscular strength and flexibility. While IG could maintain their performance in isometric muscular strength tests and increased their flexibility, CG faced a decrease in those parameters. Thus, this app-based physical exercise program had positively influenced muscular strength and flexibility in women over 60 years of age.

## Introduction

The large number of baby boomers is approaching the retirement age (Knickman and Snell, 2002) and demographic trends are leading Europe to an increasingly aging society (Grundy and Murphy, 2017). In conjunction with the general increase in life expectancy, this leads to an increase in the total number of people of advanced age. More precisely, the group of people aged 65 and over is the only one that is expected to grow in the European region (Lutz et al., 2018). Notably, this age group is at risk of physical decline if no countermeasures are taken. More than 14% of people over 60 are not able to live completely independently (WHO, 2020), that is, they are at least partially dependent on support or experience restrictions due to physical limitations. Especially women over 60 are affected by these limitations or need help in everyday situations. This is usually due to a lack of muscle strength (Doherty, 2001; Murtagh and Hubert, 2004). The bodies of those afflicted do not have the ability to function effectively and efficiently, which can also be called poor physical fitness (Corbin and Lindsey, 1997; Corbin et al., 2000). Hence, to prevent the risk of limitations in later life as a woman, it is important to train physical fitness, particularly the component of muscular strength.

Regular physical activity typically engendered through regular physical exercise can promote a high level of physical fitness and greater quality of life for older adults (Suzuki et al., 2002; Tanaka, 2009), even if this activity was started later in adulthood (Rooney, 1993). Evidence shows that a supervised, multi-component physical exercise program can have positive effects on physical fitness of women of older age (Sousa et al., 2013; Nogueira et al., 2017; De Resende-Neto et al., 2019) by improving muscular strength, balance, and flexibility, which are domains of physical fitness (Corbin et al., 2000).

To achieve a positive effect on muscle strength, and balance with physical exercise programs, a training frequency of 2–3 times per week is recommended (Nakamura et al., 2007; Carneiro et al., 2015; Jungreitmayr et al., 2021; Stojanović et al., 2021). However, improved overall physical fitness tends to show with higher training frequency (Nakamura et al., 2007; Yang et al., 2019). Findings about the impact of training frequency on physical fitness were mostly obtained after supervised training programs where frequency and the duration of the sessions were individually prescribed (Nakamura et al., 2007; Carneiro et al., 2015; Yang et al., 2019; Stojanović et al., 2021).

Although supervised exercise is often considered the superior method to deliver physical exercise (Storer et al., 2014; Fennell et al., 2016), it is not inherently better than unsupervised programs, as those have their own advantages. Unsupervised exercise programs can overcome practical barriers (Schutzer and Graves, 2004) such as transport and cost (Yardley et al., 2006; Davis et al., 2010; Kim et al., 2012) and can be integrated independently into everyday life (Geraedts et al., 2013; Müller et al., 2021). Additionally, there is preliminary evidence suggesting that unsupervised programs delivered using digital technologies (e.g., virtual reality, smartphone, or tablet apps) may have an impact on physical fitness in people of older age. (Van Het Reve et al., 2014; Park and Yim, 2016; Neumann et al., 2018; Yerrakalva et al., 2019; Gao et al., 2020; Van den Helder et al., 2020; Geraedts et al., 2021; Jungreitmayr et al., 2021; Netz et al., 2021). Findings point toward an promising but often not significant (Yerrakalva et al., 2019; Van den Helder et al., 2020; Geraedts et al., 2021), increase in lower body strength, balance, and flexibility, whereas others show significant effects on these markers (Van Het Reve et al., 2014; Park and Yim, 2016; Neumann et al., 2018; Jungreitmayr et al., 2021; Netz et al., 2021). In these studies, measurement of muscle strength, balance, and flexibility was mostly performed *via* stand-alone tests, such as chair rise (Geraedts et al., 2021; Jungreitmayr et al., 2021; Netz et al., 2021) or uni-pedal stance (Jungreitmayr et al., 2021; Netz et al., 2021), *via* short physical performance battery (Van Het Reve et al., 2014; Treacy and Hassett, 2018; Van den Helder et al., 2020) or Senior Fitness Test, respectively, (Rikli and Jones, 2013; Neumann et al., 2018).

Remarkably, positive effects on outcomes of these tests have also been demonstrated in studies that used fixed durations of exercise sessions but had the option to self-select training frequency. (Yerrakalva et al., 2019; Geraedts et al., 2021; Jungreitmayr et al., 2021; Mehra et al., 2021; Netz et al., 2021). The above-mentioned evidence suggests that unsupervised training with self-selected training frequency can improve measures of muscular strength, and balance (Geraedts et al., 2021; Jungreitmayr et al., 2021; Netz et al., 2021) and can lead to high adherence rates when it is delivered by modern technologies (i.e., tablets; Van Het Reve et al., 2014; Mehra et al., 2021).

It is important to note that adherence is a complex construct that should not be understood as mere attendance (Hawley-Hague et al., 2016). In addition to attendance, there are other measures, such as completion of the intervention, adherence to duration, as well as training intensity, that are important to consider good adherence (Hawley-Hague et al., 2016; Collado-Mateo et al., 2021). Based on this understanding, Collado-Mateo et al. (2021) identified several key factors that can positively influence adherence, with integration into daily life being one of the most important factors for older adults. Deciding when and where to exercise seems one feasible way to respect personal preferences and achieve high adherence rates. Do older people exercise enough to improve their physical fitness, or is it even more conducive to adherence, and thus outcomes, to further unlock self-selection options, such as the duration of an exercise session?

To the best of our knowledge, it has not yet been investigated whether positive effects on physical fitness and its subdomains can also be achieved, if the participants can self-select their training frequency, and session duration in an unsupervised mode.

Thus, we hypothesize that older women who receive a physical exercise program *via* a specially designed app, that allows them to determine the duration and frequency of exercise sessions, will exercise sufficiently to achieve significant effects in muscular strength, balance, and flexibility.

## Materials and Methods

### Study Design

**Figure 1:**
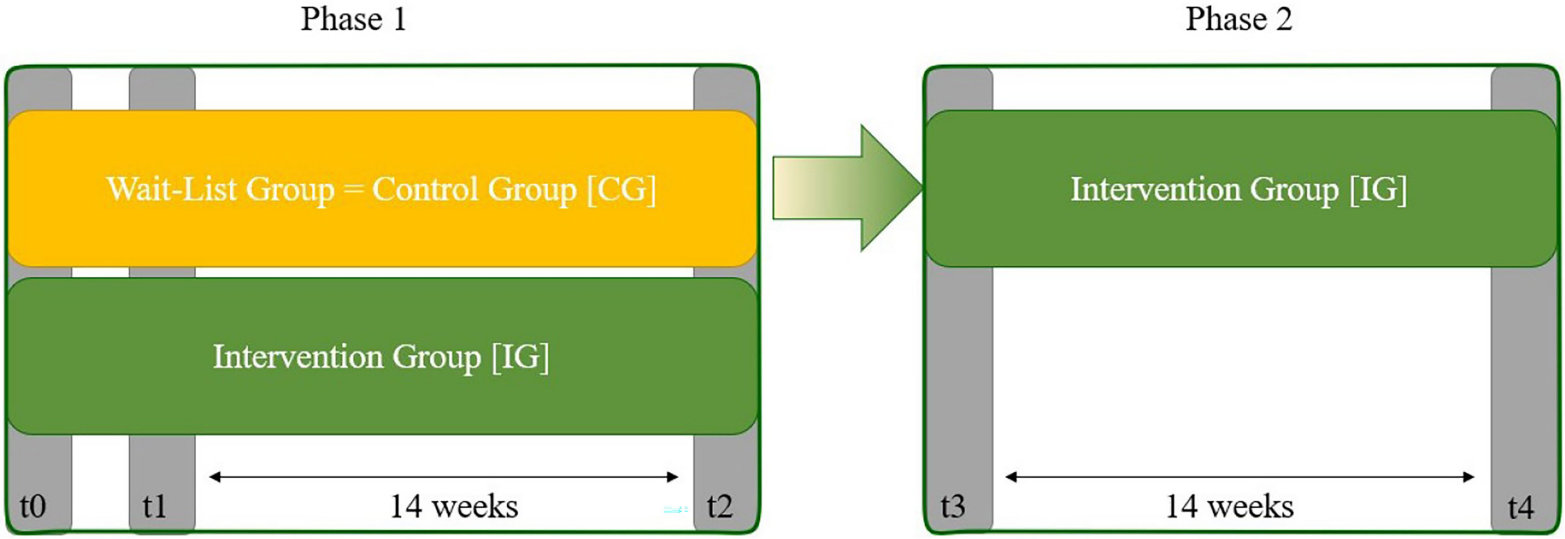
Field test design. Field test design phases; t0, getting to know the coach and assessments; t1, pre-testing for Phase 1, t2, post-testing for phase 1; t3, pre-testing for phase 2; t4, post-testing for phase 2.

The study was designed as a randomized controlled trial with a wait-list control group (CG) and has been already extensively reported elsewhere (Trukeschitz et al., 2019). For the field test of the app-based physical exercise program, the control group (CG) was offered the use of the system later (Phase 2, see Figure 1). In addition, after attending all coach appointments and scientific data collection, the CG was prospective to receive a shopping voucher (Trukeschitz et al., 2019).

The app-based physical exercise program thus consisted of a field test with two phases which is presented in Figure 1. In the first phase, both groups started with the initial assessment appointments with only the test group receiving the app-based program. The waiting group received the system after the end of the first phase and was able to test an improved version of the app in a second phase—which was conducted without a control group. This study examines the results of the first field test phase.

In the first session (t0), the participants met the coaches and were familiarized with the tests and procedures. The following appointment (t1), approximately 4 weeks later, was used to conduct the tests and hand out the hardware. After 14 weeks of intervention, the devices were collected, and the final tests were performed (t2).

### Participants

The following criteria were used for the inclusion of the study participants (see Table 1). The criteria were checked during recruitment and served as filters before randomization was performed.

**Table 1:** Study inclusion criteria.

Criterion	Measure
Age	No explicit age definition; retired for 2.5–6 years.
Activity level	Physically active individuals (PA up to 4 times per week); no concurrent physical exercise program
Physical condition	Independent, healthy individuals
Desire for activity and participation	active desire for exercise and interest in participating in a scientific study
Technical requirements	Monitor, 2–2.5m^2^ of free space, email address

#### Retirement Age

The target group consisted of people who had been retired for 2.5–6 years. Thus, no explicit age definition was used for recruitment purposes. Instead, effective retirement duration was used as a strict inclusion criterion.

#### Activity Level

Individuals were included, who were generally physically active at a maximum of four times per week, that is, subjects who were not engaged in structured exercise training. Those who attended a gym and reported being daily physically active for more than four times per week were excluded, as well as individuals who were already exercising with a personal trainer. This criterion ensured that as many women in retirement as possible were included and that the influence of a concurrent physical exercise program was avoided.

#### Physical Condition

Participants were not included when they were dependent on mobility aids such as crutches or wheelchairs. They were also not included if the suffer from any illnesses or physical ailments that could have hindered them from participating in the physical exercise program, such as rheumatism or cardiovascular disease or similar.

#### Desire for Activity and Participation

Participants should have had a desire to bring more exercise into their daily lives and were willing to participate in scientific surveys and appointments with the coach.

#### Technical Criteria

A tablet was provided to all participants to use the app-based program. A flat screen or monitor was required to use another provisioned technical component of the program, which was a feedback system offered *via* a depth imaging camera. It was also recommended that approximately 2–2.5 m^2^ of free space should be available in the home or in front of the monitor when using the depth imaging camera to perform the exercises. In addition, participants were required to have an email address.

### Recruiting and Randomization

**Figure 2:**
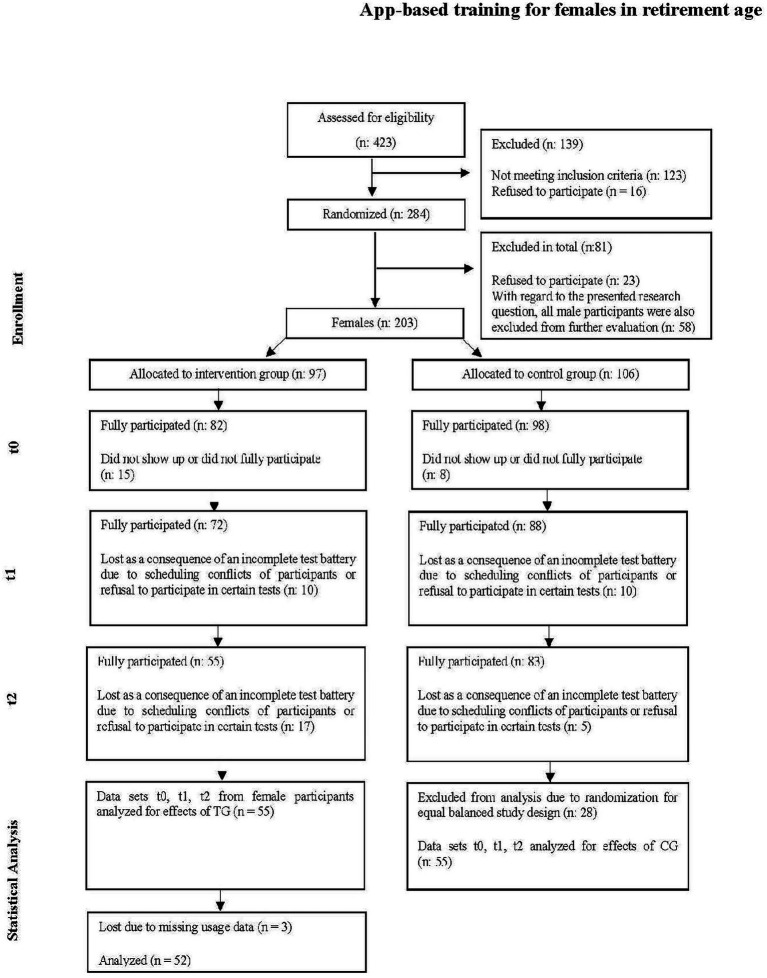
Participant flow. Flow of participants modeled on Boutron et al. (2017).

As described in Trukeschitz et al. (2019), a three-stage recruitment process before the randomization was performed in order to reach the goal of 200–250 study participants. In a first step, letters of invitation were sent out through various channels (e.g., mail, electronic newsletters, magazine and newspaper articles, postings on websites). Those interested were invited to register their wish to participate online or by telephone. A questionnaire developed for this purpose was used to verify the inclusion criteria. The items checked in this questionnaire are listed in “Participants” as well as in Table 1. Registered persons who met all criteria were included as study participants (see Figure 2). Participants were randomly assigned to IG or CG for the first field test phase and informed consent was obtained from all subjects involved in the study.

### Intervention

The app featured a physical exercise program consisting of functional exercises, a module offering suggestions for outdoor activities (e.g., hiking), e-learning courses dealing with contents on the meaning of fitness and health-enhancing training as well as an overview of the exercise achievements per day.

For the overall structure of the physical exercise program see Table 2. In the warm-up, cardiovascular stimuli, such as marching in place, were alternated with mobilization exercises, such as shoulder circles or ankle circles, with the duration of exercise set at 40 s.

**Table 2:** Structure of training sessions.

Structure	10-min session	20-min session	30-min session
Warm-up	2 sets of 3 exercises [exercises for the cardiovascular system and mobilization of joints] e.g., marching in place for 40″ shoulder circles for 40″ leg swings for 40″ repeat all three exercises	2 sets of 4 exercises [exercises for the cardiovascular system and mobilization of joints] e.g., marching in place for 40″ shoulder circles for 40″ leg swings for 40″ ankle circles for 40″ repeat all four exercises	2 sets of 5 exercises [exercises for the cardiovascular system and mobilization of joints] e.g., marching in place for 40″ shoulder circles for 40″ leg swings for 40″ ankle circles for 40″ high knee march and arm swings for 40″ repeat all five exercises
Main part	2 sets of 3 exercises [1/3 balance and 2/3 strength exercises] e.g., uni-pedal stance for 40″ chair squats for 8—12 reps table push-ups for 8—12 reps repeat all three exercises	2 sets of 6 exercises [1/3 balance and 2/3 strength exercises] e.g., uni-pedal stance for 40″ chair squats for 8—12 reps table push-ups for 8—12 reps repeat all three exercises tandem stance for 40″ lunges for 8—12 reps upright row with elastic band for 8—12 reps repeat all three exercises	2 sets of 10 exercises [1/3 balance and 2/3 strength exercises] e.g., uni-pedal stance for 40″ chair squats for 8—12 reps table push-ups for 8—12 reps repeat all three exercises tandem stance for 40″ lunges for 8—12 reps bent over row with elastic band for 8—12 reps crunch for 8—12 reps repeat all four exercises front scale variation for 40″ unilateral calf raises for 8—12 reps planks for 40″ repeat all three exercises
Cooldown	2 sets of 2 exercises [stretching exercises for upper and lower body] e.g., hip flexor stretch for 40″ triceps stretch for 40″ repeat both exercises	2 sets of 3 exercises [stretching exercises for upper and lower body] e.g., hip flexor stretch for 40″ triceps stretch for 40″ calf stretch for 40″ repeat all three exercises	2 sets of 4 exercises [stretching exercises for upper and lower body] e.g., hip flexor stretch for 40″ triceps stretch for 40″ calf stretch for 40″ neck stretch for 40″ repeat all four exercises

The main part consisted of resistance and balance exercises. Training volume per resistance exercise was set to two sets as it has been shown to be more efficient when using higher volume compared to single-sets (Kramer et al., 1997; Kraemer and Ratamess, 2004). A set designed to address muscle strength consisted of 8–12 repetitions at a rate of 3 s per repetition (Cadore et al., 2014; Izquierdo et al., 2021), with exercises that covered the entire range of motion, such as squats, lunges, or table push-ups. The strength part also included exercises with fast but safe movement, such as chair squats, to provide explosive resistance training in addition to the regular strength stimulus (Hazell et al., 2007; Izquierdo et al., 2021). Intensity for strengthening exercises was set to be at RPE 15–18 at BORG Scale, 5–7 at CR-10 scale, respectively (Williams, 2017; Izquierdo et al., 2021). Rests between sets were planned to be around 1–3 min (Izquierdo et al., 2021) and automatically generated *via* structure of the session as the exercises were organized in mini-circuits (see Table 2). Balance exercises, such as variations of uni-pedal or tandem stands were set at 40s per set (Lesinski et al., 2015). In the cooldown phase, static stretching was applied for a volume of two sets at 40s per set (Page, 2012). Accordingly, the training sessions were designed in such a way that performing the shortest session twice a week satisfied the minimum requirements in terms of muscular strength and coordination training (Garber et al., 2011; Izquierdo et al., 2021).

More detailed information about the exercise program is shown in Table 2 (i.e., overview on the structure of an exercise session) and Table 3 (i.e., overview on exercise and training variables). The aim was to create a load profile that was as homogeneous as possible while also considering individual performance (Herold et al., 2020).

**Table 3:** Load prescription.

Exercise/Training variables	Specification	Defaults
Type of exercise	Predefined	Given *via* structure (see Table 1)
Exercise duration	Predefined	8–12 repetitions OR 40 s
Exercise intensity	Recommended	RPE 5–7/vigorous intensity
Training frequency	Recommended	2–3 times per week
Training density	Self-selected	From daily to no usage
Duration of a single exercise session	Self-selected	10, 20, or 30 min

The exercise program could be accessed and viewed directly in the app on the tablet, but also used in conjunction with an exercise feedback system (Orbecc Persee) on the TV or monitor, which gave direct feedback on the correct execution of the movements (Venek et al., 2021).

The intervention started with two appointments with the coach. The first appointment (t0, see Figure 1) served to getting to know the coach, and trial procedures. The testing was explained, and familiarization tests were conducted to rule out potential learning effects. Furthermore, the concept of ratings of perceived exertion (RPE; Kilpatrick et al., 2020) was introduced so that the participants could learn to use it to assess intensities. More specifically, the CR-10 scale was used as the session RPE (Day et al., 2004; Arney et al., 2019), which allows effort to be rated in conjunction with the total load of the entire training session. Coaches anchored the scale by asking participants to recall training experiences that would provide a reference point for how the range of training intensity felt (Kilpatrick et al., 2020) The participants were instructed to perform all muscular strength exercises in vigorous (hard) intensity, using provided aids such as a ball and the elastic band as increase in strain where necessary (Colado et al., 2010; Martins et al., 2013).

After the second coach appointment (t1, see Figure 1), the coach assigned the participants of the IG to an individual difficulty level. Apart from this action, there were no differences in the treatment between the participants of the IG and CG for the course of the appointment. Another person handed out the equipment to the IG, provided information on the setup and technical use of the system. After this appointment, where they were reminded to exercise at vigorous intensities and advised to exercise at least 2–3 times per week, participants were free to decide when and where they wanted to use the system during the following 14 weeks. In addition to these choices, they could also decide for each session whether they wanted to do a 10-, 20-, or 30-min workout designed according to the latest recommendations for exercise for older people, as shown in Table 2 (Zaleski et al., 2016; Fragala et al., 2019).

In the last appointment with the coach (t2, see Figure 1), the post-testing was carried out. The participants of the IG were able to return the devices independently, so that the final appointments could be designed the same for both groups.

The study was positively evaluated by the ethics committee of the University of Salzburg (EK-GZ: 09/2018).

## Outcomes and Test Procedures

All test procedures were conducted before and after the intervention (see Figure 1).

### Lower Body Strength *via* 30CR

Sit-to-stand test are simple but good means to evaluate lower body strength (Csuka and McCarty, 1985; Guralnik et al., 1994; Bennell et al., 2011; Rikli and Jones, 2013). The 30-S Chair-Rise Test (30CR) is valid and reliable measure to evaluate lower body strength in community-dwelling adults of age (Jones et al., 1999; Rikli and Jones, 2013). The aim of this test was to complete as many stand-ups and sit-downs as possible within 30 s. The starting position was sitting on a standardized chair (~ 43 cm). The arms were held crossed at chest height. Feet were placed in hip-to-shoulder with stance. The stand-up procedure was completed when the hip and knee joints were fully extended. The sit-down procedure was completed when the buttocks fully touched the seat surface. On the command “ready, go!” this process had to be repeated as often as possible. The actions were counted to the nearest half, that is, if only standing up was completed in the last attempt, half a point was awarded for this.

### Balance *via* UPS

The ability to maintain static balance was evaluated by the uni-pedal stance test (Springer et al., 2007). The test person was asked to stand quietly on one leg for as long as possible without touching the slightly bent standing leg with the playing leg. Arms on her hips, the person had to fixate on a point on a wall at eye level. On the command of “ready, go!” the participant had to lift off one leg. Testing was performed on both sides alternately for three trials each. Times were measured with stopwatches to the nearest tenth of a second. The best of three trials was used for further analysis (Granacher et al., 2014).

### Isometric Handgrip Strength (GRIP) *via* Handheld Dynamometer

Handgrip strength has been established as an inexpensive surrogate marker for overall muscular strength (Duchowny et al., 2017; Bohannon, 2019). It was measured using a hand dynamometer (Jamar—hydraulic hand dynamometer, Sammons Preston, United States). As recommended by The American Society of Hand Therapists (Fess, 1992), the participant sits on an armchair without using the backrest and lets the hand not to be tested hang down loosely while the side to be tested is bent 90° at the elbow, with the neutral hand position facing forward. Before the measurement, the person is told that the force should be built up smoothly. On the command “ready, go,” the participant should squeeze as hard as possible. Three trials were performed for each hand while after one trial the hand was changed. The arithmetic mean of all trials was evaluated. The measurement results in values in kg (Innes, 1999; Wang et al., 2018).

### Isometric Muscular Strength Testing *via* Digital Handheld Dynamometer

Testing maximal isometric voluntary contraction with handheld dynamometry is a reliable method of testing strength of different muscle groups (Mentiplay et al., 2015; Buckinx et al., 2017) and was performed in standardized positions, all chosen so that stronger individuals could exert full force without the muscular strength of the person testing being the limiting factor (Krause et al., 2014). The test person held a mobile hand dynamometer (MicroFET2, Hoggan Scientific LLC, United States) against the measuring point of the person to be tested. The subjects were told not to apply the force in a sudden burst, but to build it up continuously within 1–2 s. The person to be tested had to start at the command “ready, go” and counteract insurmountable resistance in the position for about 4 s (Kolber and Cleland, 2005; Zander, 2018). All tests were performed on both sides alternately for three trials each. The arithmetic mean of all trials per test was evaluated. Results were recorded in kilopond. The following assessments were conducted:

#### Seated Shoulder Abduction (ShoulderF)

Shoulder abduction was measured in a seated position with the subject sitting upright on a chair without using the backrest. The arm under test had to be raised to shoulder level with 90° flexion at the elbow joint and upward pressure had to be applied against the dynamometer placed at the distal end of the upper arm.

#### Lying Hip Extension (HipExtF)

The extension of the hip was tested in prone position. The person to be tested lay on a padded table and used his hands as a pillow to avoid using her arms. The hand dynamometer was placed on the back of the thigh just after the back of the knee.

#### Side Lying Hip Abduction (HipAbdF)

Abduction in the hip joint was tested in the lateral position. The person to be tested lay on a padded table with the lower leg bent and both hands as head cushions and held the leg to be tested at hip level (Wieben and Falkenberg, 2001; Hislop et al., 2013). The hand dynamometer was placed directly above the outer ankle at the distal end of the leg.

#### Arm Flexion (BicF)

Arm flexion was measured in the supine position on a padded table. Subjects held their hand in a supinated position, elbow angle at 90°, and had to apply resistance against the device being held at the distal end of the forearm, while the untested arm had to rest beside the body.

### Flexibility *via* Range of Motion Testing

#### Lower Body (LegMob)

Straight-Leg-Raise Tests are a common measure to evaluate hamstring flexibility (Göeken and Hof, 1994; Halbertsma et al., 2001; Marshall et al., 2011; Ayala et al., 2012). In order to reliably evaluate the Straight-Leg-Raise Test, it was performed using a digital inclinometer (Van Blommestein et al., 2012).

The subjects lay supine on a padded table. The coach fixed the leg not to be tested and gently lifted the leg to be tested until the subject gave the stop signal. In this position, the angle of the leg to the lying surface was measured using an inclinometer and noted to the nearest one degree. Both legs were measured in this way.

#### Upper Body (ShoulderMob)

This test assessing shoulder flexion was performed in the supine position on a padded bench. The extended arm of the test person was brought above the head, while care was taken to ensure that the thorax remained in contact with the bench so that the amplitude of movement in the shoulder joint could actually be measured (Bartrow, 2012).

### Exercise Adherence

Exercise adherence can be described by four measures: Completion of the intervention, adherence to duration, and training intensity (Hawley-Hague et al., 2016), whereas attendance serves as singular proxy for adherence in many studies (Geraedts et al., 2014; Van Het Reve et al., 2014; Hawley-Hague et al., 2016; Van den Helder et al., 2020; Jungreitmayr et al., 2021; Mehra et al., 2021). We used frequency and duration of the app usage to operationalize adherence to the exercise program. These data were collected by using a logging software (Matomo, InnoCraft, 150 Willis St, Wellington, New Zealand) which automatically recorded usage data. The following usage features were of interest for the analysis:

#### Usage Frequency (Visit)

Frequency of usage served as a proxy for attendance. Each use of the app-based physical exercise program was defined as a visit to the app. A visit was defined as the mean usage of the app on the tablet or on the feedback system, whereby all interactions were recorded as one visit, if there were no breaks longer than 30 min. For example, if the feedback system was turned on and off multiple times within 30 min, this counted as a visit. However, if the app was used on the tablet once in the morning and again in the afternoon, this counted as two independent visits. To be able to exclude visits without actual activity, only those visits were analyzed that consisted of at least two actions. For example, if a participant opened the app on the tablet and then selected the training program this was counted as a visit. Just opening the app alone did not count as a visit.

#### Training Duration (TrgDur)

The training duration originates from the database created by the logging component. It describes the total time during the intervention phase in which the exercise program of the app was used.

#### Number of Workouts (Workouts_nr)

The workout data came from the logged data and a workout was counted as completed when the summary screen was displayed. However, it did not matter if all or only some exercises from the workout were completed. If no exercises were completed, no result was sent to the server—which means that these attempts were not counted.

#### Percentage Distribution of the Different Workout Durations (%10 Min; %20 Min; %30 Min)

As with the number of workouts, the percentage of workouts completed was taken from the logged data. For example, if the last screen of a 10-min workout was displayed and at least one of the exercises was considered completed, the workout was considered completed. In this way, all 10-, 20-, and 30-min workouts were counted. The percentage was then calculated by dividing the number of each workout group by the total number of workouts and then multiplied by 100.

### Statistical Methods

Statistical analysis was carried out using SPSS (version 27.0; IBM Corp., Armonk, NY, United States). Baseline data were described with mean ± standard deviation. To evaluate if there were any group differences at baseline, we calculated an independent samples Welch’s t-test and took Cohen’s d as the effect size. As proposed in Cohen (1988) we use the following limits to interpret effect size: 0.2 small effect, 0.5 moderate effect, 0.8 large effect.

Normal distribution of data was checked by Shapiro–Wilk’s test. As all data were distributed accordingly and did not violate Levene’s test for equality of variances, repeated measures analysis of covariances (ANCOVAs) adjusted to corresponding baseline scores were conducted to assess changes between groups over time for each fitness outcome. The level of significance was set to *p* < 0.05. Adjusted values of *p* are presented. Effect sizes are expressed as $\eta_{p}^{2}$ , whereas the effect is considered to be a small effect at 0.01, moderate at 0.06, and large at 0.14 (Cohen, 1988).

To find out whether different use of the system had an impact on the effects, the TG was divided into subgroups, and their usage behavior was analyzed descriptively. Visits were used to separate the participants into subgroups based on how often they used the system. Group intervals were defined with the Jenks natural breaks algorithm (Jenks, 1967). Thus, participants were grouped into four groups based on usage frequency, from non-users to frequent users. Frequent users used the training program 4.1–11.6 times per week (*n* = 13), occasional users 2.4–4.0 times per week (*n* = 9) rare users 1.2–2.4 times per week (*n* = 15) and non-users were logged to use below 1.2 times per week (*n* = 15).

Differences in exercise load characteristics between groups were assessed *via* ANOVA. Again, the level of significance was set to *p* < 0.05. Adjusted values of *p*are presented. Effect sizes are expressed as $\eta_{p}^{2}$ as described before (Cohen, 1988). If significant differences between subgroups were found, pairwise comparisons were performed using the Scheffé (1970) procedure.

Finally, another ANCOVA procedure was run to evaluate if fitness outcomes that significantly differed between IG and CG in general also differed between subgroups of the TG. Again the same procedure was used for the description of significance and effect sizes expressed as $\eta_{p}^{2}$ (Cohen, 1988).

## Results

### Participant Flow

After a first recruitment phase in which a total of 423 persons could be reached, 284 persons were included after applying the exclusion criteria and randomly assigned to the test or control group (see Figure 2). As 23 individuals canceled after randomization, 261 participants remained, of whom 203 were women who were thus included in this analysis. Over the course of the field test, participants were always excluded from the statistical analysis if they did not show up or showed up too late for a test appointment or did not want to complete the test battery for some other reason. It should be noted that during the study, there were no discontinuations due to injuries from using the system or the like. Furthermore, no adverse side effects were reported. For the statistical evaluation, 55 data sets of the TG were available at the end of the first field test phase (see Figure 1). To obtain a balanced evaluation design, 55 data sets were randomly drawn from the 83 data sets of the CG. For the detailed evaluation of the subgroups of the TG, another 3 missing had to be accepted, since usage data were missing here. Therefore, these evaluations were carried out with 52 instead of 55 data sets.

### Baseline Data

Table 4 shows that with a mean of 16.6 (± 3.3) and 15.7 (± 4.7) chair-rise repetitions in IG and CG respectively, the participants scored well above the norm values for women within the age group of 60–64 years (14.5 ± 4.0) as well as for those within 65–69 years of age (13.5 ± 3.5) indicating a very good physical fitness regarding lower body strength (Rikli and Jones, 1999). Overall muscular strength represented *via* handgrip strength (IG: 26.5 ± 4.8; CG 26.5 ± 4.9) also showed above average fitness regarding this parameter compared to norm values for both age groups 60–64 (23.6 ± 6.5) and 65–69 (22.1 ± 6.6) respectively (Wang et al., 2018). Balance assessed *via* Uni-Pedal Stance (UPS) showed a highly above ability compared to the norm values of women between 60 to 69 years of age with 30.4 (± 16.4), being 50.8 (± 18.0) in IG and 52.3 (± 17.5) in CG (Springer et al., 2007). Range of motion tests showed good flexibility in both shoulder and hip joint, whereby the maximum limits of 180° in the shoulder joint and 140° in the hip joint (Bartrow, 2012) were not reached in both IG (shoulder 168.8 ± 7.3, leg 98.0 ± 16.1) and CG (shoulder 171.4 ± 6.7; leg 99.7 ± 16.9) at baseline.

**Table 4:** Baseline data of test and control group.

Baseline data	IG (n: 55)		CG (n: 55)		Welch’s *t*-test				95% CI of the differences	
	*M*	SD	*M*	SD	*t*	df	*p*	Cohen’s *d*	Lower	Upper
Age, yrs	65.4	1.5	65.2	1.5	0.698	107.804	0.487	1.503	−0.368	0.768
t1_30CR, cts	16.6	3.3	15.7	4.7	1.285	97.612	0.202	0.245	−0.5	2.5
t1_UPS, s	50.8	18.0	52.3	17.5	−0.449	107.913	0.655	0.086	−8.2	5.2
t1_Grip, kg	26.5	4.8	26.5	4.9	−0.045	107.981	0.964	0.009	−1.9	1.8
t1_ShoulderF, kg	11.6	2.7	11.2	2.0	0.900	99.664	0.370	0.172	−0.5	1.3
t1_HipExtF, kg	15.0	3.0	15.6	4.1	−0.949	98.546	0.345	0.181	−2.0	0.7
t1_HipAbdF, kg	10.8	2.4	10.5	2.5	0.584	107.764	0.560	0.111	−0.6	1.2
t1_BicF, kg	14.9	2.5	16.1	3.1	−2.261	104.072	0.026	0.431	−2.3	−0.1
t1_ShoulderMob	168.8	7.3	171.4	6.7	−1.905	107.175	0.059	0.363	−5.2	0.1
t1_LegMob	98.0	16.1	99.7	16.9	−0.527	107.721	0.600	0.100	−7.9	4.6

*t1, baseline test date; 30CR, 30-s Chair-Rise Test expressed in counts (cts); UPS, Uni-Pedal Stance Test expressed in seconds (s); Grip, Handgrip Strength Test expressed in kilogram (kg)—as are all isometric strength measurements; ShoulderF, isometric strength testing for shoulder abduction; HipExtF, isometric strength testing for prone hip extension; HipAbdF, isometric strength testing for side lying hip abduction; BicF, isometric strength testing for elbow flexion; ShoulderMob, range of motion testing for shoulder flexion expressed in degrees; LegMob, range of motion testing for hip flexion in lying supine position expressed in degrees*.

Baseline data (see Table 4) showed a statistically significant difference with moderate effect size between groups in isometric muscular strength when flexing the elbow joint, but in no other outcome criteria. To account for baseline differences further calculations regarding effects over time between groups were adjusted to baseline (t1) values.

### Outcomes and Estimation

The findings show positive results on the physical fitness of the IG compared to the TG. Univariate repeated measures analyses of covariances show statistically significant differences between groups in isometric strength at hip extension, hip abduction, elbow flexion, and in both parameters regarding range of motion testing. The analyses showed moderate effect sizes on all results, except for the difference in elbow flexion isometric force, which indicated a large effect (see Table 5).

**Table 5:** Effects over time between test and control group.

	*IG*/*n:* 55 Δ*M*	*CG*/*n:* 55 Δ*M*	SE	ANCOVA			95% CI of the differences	
				*p*	*F* (1, 107)	$\eta_{p}^{2}$	Lower	Upper
30CR, cts	0.118	0.673	0.312	0.213	1.573	0.014	−0.716	0.161
UPS, sec	1.827	1.839	0.993	0.933	0.000	0.000	−1.398	1.386
Grip, kg	0.875	1.403	0.329	0.259	1.289	0.012	−0.725	0.197
ShoulderF, kg	0.214	−0.504	0.301	0.095	2.832	0.026	−0.064	0.783
HipExtF, kg	−0.007	−2.19	0.491	0.002	9.833	0.084	0.402	1.782
HipAbdF, kg	0.292	−0.692	0.257	0.008	7.332	0.064	0.132	0.853
BicF, kg	0.641	−1.798	0.365	0.001	21.827	0.169	0.702	1.737
ShoulderMob	2.601	0.407	0.661	0.022	5.421	0.048	0.163	2.032
LegMob	7.269	0.870	1.260	0.001	12.875	0.107	1.432	4.967

*Repeated measures analysis of covariance adjusted to baseline; Δ, mean difference t2−t1 within group; 30CR, 30-s Chair-Rise Test expressed in counts (cts); UPS, Uni-Pedal Stance Test expressed in seconds (s); Grip, Handgrip Strength Test expressed in kilogram (kg)—as are all isometric strength measurements; ShoulderF, isometric strength testing for shoulder abduction; HipExtF, isometric strength testing for prone hip extension; HipAbdF, isometric strength testing for side lying hip abduction; BicF, isometric strength testing for elbow flexion; ShoulderMob, range of motion testing for shoulder flexion expressed in degrees; LegMob, range of motion testing for hip flexion in lying supine position expressed in degrees*.

The descriptive analysis of subgroups considering usage of the program provides an overview regarding exercise adherence markers (see Table 6). Pairwise comparisons showed a significant difference in number of workouts between all groups and a significant difference in training duration between all groups, except between occasional and rare users with a *p* = 0.381, 95% CI [−2.1; −11.2].

**Table 6:** Descriptive analysis of subgroups considering usage of the exercise program.

	Frequent users/n: 13		Occasional users/n: 9		Rare users/n: 15		Non-users/n: 15		ANOVA		
	*M*	*SD*	*M*	*SD*	*M*	*SD*	*M*	*SD*	*p*	F(3/48)	$\eta_{p}^{2}$
Age, yrs	65.0	1.6	65.1	1.3	65.8	1.7	65.9	1.8	0.354	1.110	0.065
Trg. Dur, h	24.7	8.3	15.0	7.0	10.4	4.5	3.2	2.3	0.001	32.991	0.673
Workouts, n	65.3	18.7	41.3	15.3	23.5	6.7	8.8	5.7	0.001	54.342	0.773
%10 min	43.6	20.8	52.1	30.3	41.5	29.5	54.8	38.2	0.606	0.619	0.037
%20 min	34.7	22.0	23.6	17.0	26.1	17.8	28.3	37.4	0.755	0.398	0.024
%30 min	21.8	25.2	24.3	27.2	32.5	36.1	16.9	31.5	0.579	0.662	0.040

*Frequent users used the physical exercise program 4.1–11.6 times per week; occasional users 2.4–4.0 times per week; rare users 1.2–2.4 times per week; non-users: below 1.2 times per week; Trg. Dur, total duration of training within the intervention period expressed in hours; Workouts, total number (n) of workouts completed within the intervention period; %10 min, percentage of 10-min training sessions over the entire intervention period; %20 min and %30 min, corresponding to the same measure as for %10 min*.

To assess if usage frequency led to different results, univariate ANOVAs were calculated for the subgroups, as can be seen in Table 7.

**Table 7:** Effects over time between subgroups.

	Frequent users/n: 13		Occasional users/n: 9		Rare users/n: 15		Non-users/n: 15		ANOVA		
	*M*	*SD*	*M*	*SD*	*M*	*SD*	*M*	*SD*	*p*	*F* (3/48)	$\eta_{p}^{2}$
Δ_HipExtF, kg	−0.469	3.922	0.633	3.420	1.527	3.131	−0.733	2.641	0.236	1.464	0.084
Δ_HipAbdF, kg	0.239	1.239	0.478	1.506	0.773	1.123	0.047	1.241	0.441	0.915	0.054
Δ_BicF, kg	0.646	2.642	0.611	2.418	1.213	2.020	0.733	3.021	0.921	0.163	0.010
Δ_ShoulderMob	3.792	3.867	1.150	3.034	2.120	4.399	3.857	4.789	0.339	1.150	0.067
Δ_LegMob	8.439	11.028	6.033	7.198	6.610	7.847	6.643	9.933	0.922	0.162	0.010

*Frequent users used the physical exercise program 4.1–11.6 times per week; occasional users 2.4–4.0 times per week; rare users 1.2–2.4 times per week; non-users: below 1.2 times per week; univariate analysis of variance using Δ (t2−t1); HipExtF, isometric strength testing for prone hip extension; HipAbdF, isometric strength testing for side lying hip abduction; BicF, isometric strength testing for elbow flexion; ShoulderMob, range of motion testing for shoulder flexion expressed in degrees; LegMob, range of motion testing for hip flexion in lying supine position expressed in degrees*.

Subgroup analysis showed that despite presenting significantly different duration of training, number of workouts (see Table 6) there were no significant differences between the usage-based subgroups of the TG (see Table 7).

## Discussion

With our study, we investigated the effects on an app-based physical exercise program on physical fitness. Our main findings imply that participants who used the app-based physical exercise program had significantly positive effects on muscle strength and flexibility compared to the control group.

Our sample consisted of women over 60 years of age who, compared with their peers, could be considered fit. As our data show, the adherence rate (in terms of attendance) was 71%, which means 37 of 52 participants used the exercise program at least 1.2–2.4 times per week. This adherence rate exceeds general web-based interventions, which have on average 50% and once-weekly use (Kelders et al., 2012), as well as typically reported rates for exercise programs, often with half of participants quitting (Chao et al., 2000; Picorelli et al., 2014). The use of new technology in the current study (i.e., tablets) might have led to the relatively high adherence rates in our study which is in line with observations of previous studies utilizing modern technologies to deliver physical exercise programs (Valenzuela et al., 2018; Mehra et al., 2021). Additionally, the autonomy to freely chose the duration of a single exercise session and training frequency which can allow for a better integration of physical exercises into everyday life, could be another reason for the good adherence (Collado-Mateo et al., 2021) as we were able to achieve slightly better adherence rates than in programs where session duration was fixed, which achieved adherence rates of 60–69% among completers (Geraedts et al., 2014; Jungreitmayr et al., 2021; Mehra et al., 2021).

Our results invite a nuanced consideration of the effects of an app-based physical exercise program for women aged 60 years and older, as functional performance of lower body strength, handgrip strength, and balance remained unchanged, whereas muscular strength and range of motion tests showed significant improvements in IG compared with CG. At first glance, these results are unexpected, as the exercise program was aligned with current recommendations (Garber et al., 2011; Cadore et al., 2014; Fragala et al., 2019; Izquierdo et al., 2021) and contained all the necessary stimuli, such as training in repetition ranges from 8 to 12, fast movements and recommended training intensities from moderate to demanding, all which were found to produce positive effects on physical fitness, as well as its components, in older adults (Bemben et al., 2000; Hunter et al., 2001; Kalapotharakos et al., 2005; Richardson et al., 2019; Herda et al., 2020). Furthermore, the usage data also show that the exercise frequency was well within in a range that should be able to produce effects, since even one or two sessions per week have been proven able to do this (Taaffe et al., 1999; Richardson et al., 2019; Jungreitmayr et al., 2021; Stojanović et al., 2021). While neither the frequency of use nor the composition of the program can be considered a reason for the lack of effect on lower body strength and handgrip strength, the testing procedure itself as well as the uncertainty whether the participants have reached the appropriate exercise intensity (due to the unsupervised mode of the exercise program) remain possible reasons for these results. It should be considered that the participants in the 30-s Chair-Rise Test have already started with a very good result and the test, although it could show good discrimination between highly active and low active individuals (Jones et al., 1999), may not discriminate sufficiently within this active population, which means that a significant improvement of the already high level may not be noticed using this test.

Furthermore, a lack of an appropriate intensity remains a possible explanation. Even though many different intensities can be used successfully (Bemben et al., 2000; Hunter et al., 2001; Kalapotharakos et al., 2005), an actual moderate to high exercise intensity, achieved *via* the repetition velocity of an exercise, seems to be advisable in order to achieve an improvement in the chair standing tests (Alexander et al., 2001). Although exercise intensity was prescribed *via* rating of perceived exertion, it was up to the participants to actually achieve this intensity and there is a possibility that exercises were done at other intensities (Kilpatrick et al., 2020). Evidence exists that self-selected exercise intensity is often below the recommended one (Focht, 2007; Elsangedy et al., 2013; Dias et al., 2018). Notably, recent studies dealing with unsupervised technology-based programs have faced similar challenges, with chair-rise tests producing promising but not significant results (Yerrakalva et al., 2019; Van den Helder et al., 2020; Geraedts et al., 2021). Hence, we conclude that self-selected exercise intensity of a home-based training causes lower effects compared to a supervised training (Thiebaud et al., 2014; Lacroix et al., 2017). This holds especially true when studying the effects on physical fitness in healthy participants by means of chair-rise test (Lacroix et al., 2017).

Regarding balance ability, it must be noted that this was already set at such a high level compared to age-specific norm values at the start of the intervention that no further improvement in this area could be expected. This is all the more important as an increase in UPS should only be considered as an actual increase if the improvement exceeds 24 s (Goldberg et al., 2011).

One of the strengths of this study is, that physical fitness was measured with additional test besides those commonly used in similar studies, that is, sit-to-stand, and uni-pedal stance test, to ensure a detailed look at the effects of the program evaluated.

Looking at the significantly changed outcomes, it is evident that significant differences between IG and CG were achieved in isometric strength as well as range of motion. Differences between IG and CG show that the IG increased isometric strength to a little extent but more important we can notice a decrease in CG. The increase in muscular strength could be due to the fact that positive adaptations in healthy elderly subjects can be expected even at low intensities (Taaffe et al., 1996; Takarada and Ishii, 2002; Watanabe et al., 2014). Regarding the developments in CG, it can be said that decreases in the domain of isometric strength within 3 months are highly likely and can also be found in other studies (Henwood and Taaffe, 2008; Park and Yim, 2016). In particular, when physical activity is lacking, as reported by Doherty (2001), muscle strength decreases significantly, becoming functionally important in women in the 7th decade of life, which is consistent with findings on the loss of muscle strength in inactive people of age (Goodpaster et al., 2006; Forrest et al., 2007; Henwood and Taaffe, 2008).

The significant increase in shoulder and hip joint range of motion can be attributed in part to the fact that flexibility can benefit from resistance training (Barbosa et al., 2002; Monteiro et al., 2008; Carneiro et al., 2015) as well as stretching as applied in the program (Bandy et al., 1997; Feland et al., 2001; Batista et al., 2009). As far as stretching is concerned, it can be assumed that, in addition to the novelty of the exercises, the intensity may have been more appropriate, since the feeling of exertion during stretching is often difficult to assess subjectively, that is, no overload or heavy strain might have been noticed (Lim and Park, 2017).

Surprisingly, no significant differences in the outcomes that have been influenced by the intervention were found with the evaluation of the IG subgroups, which indicates that the results within the domains of muscular strength, and flexibility are not linked onto frequency of exercise or the duration of it.

The results of this study should be interpreted considering the following limitations. Since we did not assess rating of perceived exertion after each session to control for the exercise intensity, it remains the possibility that the recommended exercise intensity was not achieved by all participants. To improve comparability and reproducibility, we recommend that further exercise studies using modern technology (e.g., smartphone- or tablet-based apps) report both markers of external load and internal load to appropriately characterize the exercise intensity (Gronwald et al., 2019; Herold et al., 2020). Given that the duration of the intervention is an important factor contributing to its success (Silva et al., 2014), the short intervention duration of 14 weeks could be another potential confounder. Lastly, our findings can be only generalized to the group of very fit women over 60 years of age, whereas the effects might be even more pronounced in older and/or less fit participants given the promising findings of another study in the group of frail older adults (Jungreitmayr et al., 2021).

### Take Home Message

Since the adherence to our app-based physical exercise program was relatively high, physical exercise programs delivered by modern technologies could be a promising option allowing older individuals to better incorporate physical exercise and physical activity in their everyday life, thus make a valuable contribution to prevent (slow down) the age-related decline of muscular strength or range of motion. Our findings suggest that our app-based physical training program is a promising option to counteract the decline in physical fitness and mobility and thus is well-situated to enable women over 60 years to achieve a physically active lifestyle.

## Data Availability Statement

The datasets presented in this article are not readily available because of data privacy regulations. Requests to access the datasets should be directed to sonja.jungreitmayr@plus.ac.at.

## Author Contributions

SJ: conceptualization, formal analysis, investigation, writing—original draft preparation, and visualization. SJ (data of all testing carried out in practice), CK, and VV (usage data): data curation. SR-D and SJ: writing—review and editing. SR-D: supervision. VV: project administration and funding acquisition. All authors have read and agreed to the published version of the manuscript.

## Funding

The co-operative project, “Fit in einen neuen Lebensabschnitt mit neuen Technologien—AAL-Testregion Salzburg/Wien (fit4AAL)” with the grant number 862035, was funded by the Austrian Federal Ministry for Transport, Innovation and Technology under the research program benefit.

## Conflict of Interest

CK and VV were employed by company Salzburg Research Forschungsgesellschaft mbH. SJ is the owner of MyBodyCoach.

The remaining authors declare that the research was conducted in the absence of any commercial or financial relationships that could be construed as a potential conflict of interest.

## Publisher’s Note

All claims expressed in this article are solely those of the authors and do not necessarily represent those of their affiliated organizations, or those of the publisher, the editors and the reviewers. Any product that may be evaluated in this article, or claim that may be made by its manufacturer, is not guaranteed or endorsed by the publisher.
